# Insulin receptor endocytosis in the pathophysiology of insulin resistance

**DOI:** 10.1038/s12276-020-0456-3

**Published:** 2020-06-23

**Authors:** Catherine Hall, Hongtao Yu, Eunhee Choi

**Affiliations:** 1 Department of Pathology and Cell Biology, Vagelos College of Physicians and Surgeons, Columbia University, 630 West 168th Street, New York, NY 10032 USA; 2 Laboratory of Cell Biology, School of Life Sciences, Westlake University, Hangzhou, Zhejiang 310024 China; 3 Department of Pharmacology, University of Texas Southwestern Medical Center, 6001 Forest Park Road, Dallas, TX 75390 USA

**Keywords:** Endocytosis, Mechanisms of disease, Homeostasis

## Abstract

Insulin signaling controls cell growth and metabolic homeostasis. Dysregulation of this pathway causes metabolic diseases such as diabetes. Insulin signaling pathways have been extensively studied. Upon insulin binding, the insulin receptor (IR) triggers downstream signaling cascades. The active IR is then internalized by clathrin-mediated endocytosis. Despite decades of studies, the mechanism and regulation of clathrin-mediated endocytosis of IR remain incompletely understood. Recent studies have revealed feedback regulation of IR endocytosis through Src homology phosphatase 2 (SHP2) and the mitogen-activated protein kinase (MAPK) pathway. Here we review the molecular mechanism of IR endocytosis and its impact on the pathophysiology of insulin resistance, and discuss the potential of SHP2 as a therapeutic target for type 2 diabetes.

## Insulin: Keeping the receptors signaling

A potential cancer treatment also shows promise for treatment of type 2 diabetes. When insulin receptors (IRs) on cell surfaces bind to insulin, they send out signals that trigger glucose uptake, lowering blood sugar. The duration of IR signaling is crucial for metabolic health, but its regulation is poorly understood. Eunhee Choi, Columbia University, New York, and Hongtao Yu, Westlake University, Hangzhou, and a coworker have reviewed how IR signaling is controlled. They report that inhibiting the protein SHP2 may prolong IR signaling and improve how the body responds to insulin. Because SHP2 is also implicated in cancer, inhibitors have already been developed and could be tested for treatment of metabolic diseases. These results illuminate the fundamentals of a key metabolic pathway, and may help in treatment of type 2 diabetes.

## Introduction

The pancreatic hormone insulin controls the metabolism of glucose and lipids in our body^1,2^. It promotes glucose uptake and its conversion into glycogen and lipids for energy storage in metabolic tissues, thereby enabling the maintenance of proper blood glucose levels. Normal circulating insulin levels are necessary for glucose homeostasis. Persistent hyperinsulinemia, an above normal level of insulin in the blood, is associated with insulin resistance. Insulin resistance is a hallmark of metabolic diseases, including type 2 diabetes and atherosclerosis^1–6^. Understanding the mechanisms of insulin resistance is therefore essential for the continued development of effective therapeutic strategies to treat these prevalent diseases.

The relationship between hyperinsulinemia and insulin resistance is complicated. The prevailing view is that the pancreas produces more insulin to compensate for the rise in blood glucose level caused by defective insulin signaling^7–10^. An alternative view is that hyperinsulinemia may initiate and expand insulin resistance^11–14^. These are not mutually exclusive concepts and probably act in parallel.

At the cellular level, insulin binds to the insulin receptor (IR) on the plasma membrane (PM) and triggers the activation of signaling cascades to regulate metabolism and cell growth. Following activation, insulin-bound IR can be internalized by clathrin-mediated endocytosis (CME)^15–18^. As a key CME adaptor, the assembly polypeptide 2 (AP2) complex links clathrin to both the cargo and lipids on the PM. The AP2 complex has four subunits: AP2A, AP2B1, AP2M1, and AP2S1. It has a large globular core consisting of the entirety of both AP2M1 and AP2S1 subunits, along with the N-terminal trunk domain of AP2A and AP2B1. The AP2 core recognizes sorting signals from the cargo, such as di-leucine and YXXΦ (X, any amino acids; Φ, hydrophobic residues) motifs. The C-terminal appendages of the AP2A and AP2B1 subunits extend from the core and bind to clathrin and other accessory proteins, thus promoting clathrin vesicle formation. The endocytosis of the IR–insulin complex is a key mechanism that regulates the intensity and duration of insulin signaling. In contrast, persistent hyperinsulinemia may accelerate IR endocytosis, thus decreasing the functional IR level at the PM. Biochemical and immunohistochemistry studies have shown that the level of IR at the PM might be reduced in diabetes patients^19–21^. These findings suggest that reduced IR levels at the PM might be a contributing factor to insulin resistance in human patients.

The spindle checkpoint ensures accurate chromosome segregation and prevents aneuploidy^16,22–27^. MAD2 and BUBR1 are critical spindle checkpoint proteins. In response to unattached kinetochores, they bind to CDC20 and BUB3 to form the mitotic checkpoint complex (MCC). MCC prevents chromosome segregation by directly inhibiting the anaphase-promoting complex/cyclosome^28–34^. When all kinetochores are attached to the bipolar spindle, p31^comet^ binds to active MAD2 and inactivates the spindle checkpoint^35–39^.

**Fig. 1:**
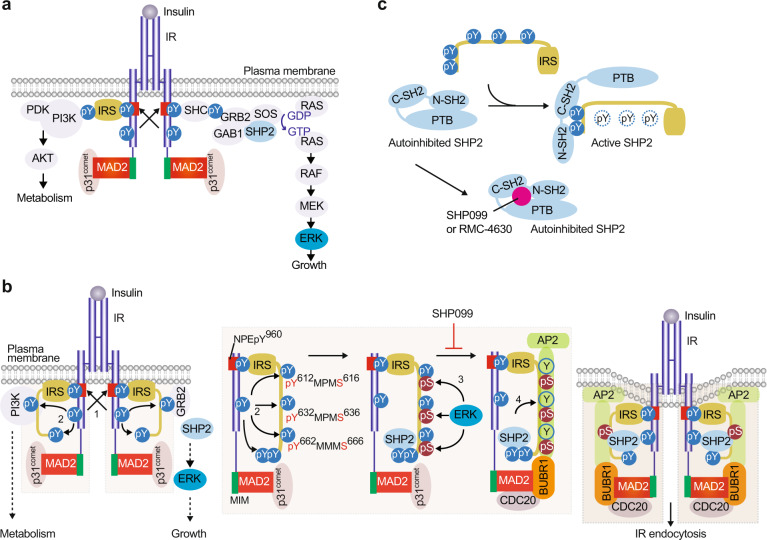
IRS- and MAD2-dependent mechanisms collaborate to trigger IR endocytosis. **a** The insulin receptor signaling pathway. **b** Model of the regulation of IR endocytosis by IRS and spindle checkpoint proteins. In the basal state, p31^comet^ prevents IR endocytosis. Activated IR auto-phosphorylates multiple tyrosine residues on IR, including Y960 in the NPEY^960^ motif (1), recruits IRS proteins and initiates insulin signaling cascades. IR phosphorylates Y612/Y632/Y662 of the YXXΦ motifs on IRS1 (2). Activated ERK phosphorylates S616/S636/S666 on IRS1 (3). SHP2 binds to the C-terminal phosphotyrosine sites on IRS1 and dephosphorylates pY612/pY632/pY662 of the doubly phosphorylated IRS1 (pY/pS) to facilitate the IRS1–AP2 interaction (4). IR-bound MAD2 binds to BUBR1-CDC20, providing another binding surface for AP2. These two modules promote IR endocytosis. Inhibition of the feedback regulation prevents IR endocytosis, prolongs metabolic branch of insulin signaling, and improves insulin sensitivity. MIM, MAD2-interacting motif.

Our previous studies have revealed an unexpected function of the spindle checkpoint in insulin signaling through regulating IR endocytosis. MAD2 binds to the C-terminal MAD2-interacting motifs (MIMs) of IR and recruits AP2B1 to IR through BUBR1-CDC20^16,21,40^ (Fig. 1a, b). p31^comet^ prevents IR endocytosis by inhibiting the interaction between BUBR1-CDC20-AP2 and IR-bound MAD2. Liver-specific *p31*^*comet−/−*^ mice have reduced IR levels on the PM of hepatocytes and develop whole-body insulin resistance^40^. Conversely, BUBR1 deficiency delays insulin-mediated IR endocytosis and improves insulin sensitivity in mice^40,41^. These findings suggest that the dysregulation of IR endocytosis is a potential mechanism underlying insulin resistance.

It has long been known that IR kinase activity is crucial for receptor endocytosis^42,43^, suggesting that IR endocytosis normally occurs after the receptor has been activated and has transduced signals downstream. However, how activated IR is selectively internalized remained largely unknown until our recent study. We have discovered a regulatory feedback mechanism of IR endocytosis through the SHP2–MAPK pathway^21^. Inhibition of this regulatory feedback delays IR endocytosis, prolongs metabolic signaling, and improves insulin sensitivity. Here we review this newly discovered regulatory mechanism of IR endocytosis, discuss its impact on pathophysiology, and highlight the key unanswered questions.

## The SHP2–MAPK pathway in metabolic regulation

IR is a receptor tyrosine kinase (RTK) that is activated by insulin binding^44^. The binding of multiple insulin molecules to an IR destabilizes its autoinhibitory conformation, leading to its trans-autophosphorylation and activation^45–47^. The tyrosine-phosphorylated IR recruits and phosphorylates IR substrate (IRS) or SRC homology 2 domain-containing (SHC) proteins at several tyrosine residues^48,49^ (Fig. 1a). These tyrosine phosphorylation events recruit additional effectors and activate two major signaling cascades: (1) the phosphatidylinositol 3-kinase (PI3K)–protein kinase B/AKT (PI3K–PKB/AKT) pathway and (2) the MAPK pathway. The PI3K–PKB/AKT pathway is primarily responsible for controlling metabolism. The MAPK pathway mainly controls cell growth and proliferation. Accumulating evidence now suggests that the dysregulation of insulin-mediated MAPK pathway activation may contribute to insulin resistance^50–53^.

The phosphorylated IRS and SHC proteins bind to growth factor receptor-bound protein 2 (GRB2) and then recruit the guanine nucleotide exchange factor, son of sevenless (SOS), to activate the RAS–MAPK pathway^54^. SHP2, encoded by *PTPN11*, is a nonreceptor protein tyrosine phosphatase (PTP) and a scaffolding protein that controls SOS2-mediated MAPK pathway activation. SHP2 contains two tandem SH2 domains (N-terminal SH2 and C-terminal SH2), a PTP domain, and a C-terminal tail^55^ (Fig. 1c). In the basal state, the SH2 domains engage the catalytic pocket in the PTP domain and sterically block the active site. Upon insulin stimulation, the two SH2 domains in SHP2 interact with the phosphotyrosine sites in IRS proteins and GRB2-associated binder protein 1 (GAB1), thus breaking the autoinhibitory interface and rendering the active site available for substrates^56,57^. The phosphatase activity of SHP2 is required for the formation of the GAB1–GRB2–SOS1 complex, which in turn promotes RAS activation^58^ (Fig. 1a). Activated RAS binds to RAF and causes RAF translocation to the PM. RAF then activates the dual-specificity serine and threonine kinases, MEK1 and MEK2, which phosphorylate and activate extracellular signal-regulated kinase 1 and 2 (ERK1 and ERK2)^59^.

ERK1/2 are the best-characterized MAPK family members. ERK1/2 phosphorylate serine or threonine residues that are followed by a proline residue (S/T-P). Over 200 substrates of ERK1/2, including SREBP1, SREBP2^60,61^, and peroxisome proliferator-activated receptor γ^52^, have been identified so far^62–64^. ERK1 and ERK2 share 75% amino acid identity and phosphorylate the same substrates with similar specificity in vitro. However, *ERK1*^*−/−*^ mice are viable and fertile, but *ERK2*^*−/−*^ mice are not viable^65–68^, suggesting that these kinases are not redundant and have tissue-specific roles. Although *ERK1*^*−/−*^ mice have been shown to be more sensitive to insulin, diet-induced obesity mice and leptin-deficient (*ob/ob*) mice have elevated ERK activity^51,69,70^. Pharmacological inhibition of ERK improves insulin sensitivity in both diet-induced obesity and *ob/ob* mice^52^. Furthermore, the basal activity of ERK is elevated in human type 2 diabetes^71–73^, indicating that the MAPK pathway may be a potential therapeutic target for insulin resistance and metabolic disorders.

SHP2 is the first reported oncogenic tyrosine phosphatase. As an upstream regulator of the MAPK pathway, SHP2 promotes cell growth and proliferation. Conventional *SHP2*^*−/−*^ mice are embryonic lethal^74^. Tissue-specific *SHP2*^*−/−*^ mice survive and show that SHP2 controls metabolic homeostasis in multiple tissues. For example, striated and cardiac muscle-specific *SHP2*^*−/−*^ mice display severe dilated cardiomyopathy, undergo premature death, and exhibit insulin resistance^75^. Neuronal SHP2 dysfunction causes early-onset obesity accompanied by high levels of leptin, insulin, glucose, and triglycerides^76^. On the other hand, liver-specific *SHP2*^*−/−*^ mice exhibit enhanced insulin sensitivity^77,78^. Pharmacological inhibition of SHP2 markedly increased glucose and insulin sensitivity in a diet-induced obesity mouse model^21^. The introduction of adeno-associated viruses encoding SHP2 short-hairpin RNAs into the liver confirms a role of SHP2 in metabolic homeostasis in mice^21^. In addition, deficiency of GAB1, the binding partner of SHP2, in the liver exhibits improved insulin sensitivity, together with enhanced AKT and blunted MAPK pathway activation^79^. This finding suggests that the SHP2–MAPK pathway may offset certain aspects of insulin signaling in the liver, thus prolonging the metabolism signaling branch and improving whole-body insulin sensitivity.

### The SHP2–MAPK pathway in IR endocytosis

How does the SHP2–MAPK pathway control metabolism? What are the main targets of SHP2 and MAPK in this pathway? IRS proteins are crucial adaptors that transduce signals from IR on the PM to intracellular downstream effectors and adaptors^48^. Insulin-activated IR phosphorylates its own NPEY^960^ motif in the juxtamembrane domain (Fig. 1a, b). The phosphotyrosine-binding domain of IRS proteins directly binds to the phosphorylated NPEY^960^ motif in IR^80–84^. The NPEY^960^ motif in IR had been implicated in AP2 binding and in IR endocytosis, but the mechanism remained unclear^85,86^. Activated IR phosphorylates several tyrosine residues in the IRS proteins, including multiple YXXΦ motifs in the middle region (Fig. 1b)^87,88^. These phosphotyrosine motifs interact with PI3K, facilitating the activation of the PI3K–PKB/AKT pathway. SHP2 binds directly to the C-terminal phosphotyrosine residues in IRS proteins and dephosphorylates the tyrosine residues in the YXXΦ motifs, thus negatively regulating PI3K activity^89,90^ (Fig. 1b, c). Multiple serine and threonine residues in IRS proteins can also be phosphorylated upon insulin stimulation^91^. The increased serine/threonine phosphorylation of IRS proteins is associated with insulin resistance in human and mouse models. ERK is one of the most well-known kinases of IRS proteins. Strikingly, the serine residues that follow the YXXΦ motifs are phosphorylated by ERK^50,92^. ERK-mediated phosphorylation of IRS proteins has been shown to reduce their tyrosine phosphorylation through negative feedback^50,91,92^.

We have recently shown that IRS1 and IRS2 bind directly to the clathrin adaptor AP2M1 through multiple YXXΦ motifs and promote insulin-activated IR endocytosis^21^ (Fig. 1b). Interestingly, these AP2M1-YXXΦ interactions are regulated by a phosphorylation switch mediated by ERK and SHP2. ERK-mediated serine/threonine phosphorylation promotes SHP2-mediated tyrosine dephosphorylation of the YXXΦ motifs, resulting in a switch from phosphotyrosine to phospho-serine/threonine. Only phospho-serine/threonine-containing IRS proteins can interact with AP2 and trigger IR endocytosis.

**Fig. 2:**
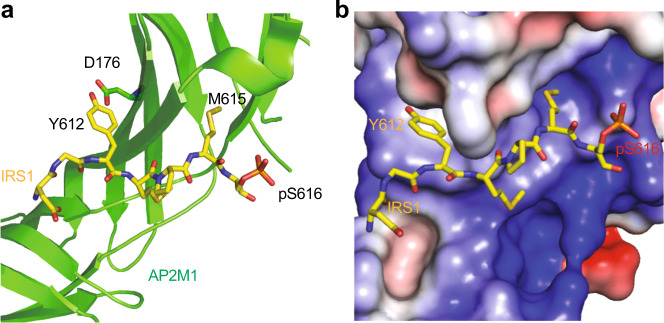
Structure of AP2M1 bound to pS616-IRS1. **a** Ribbon diagram of the crystal structure of AP2M1-pS616-IRS1 (PDB ID: 6BNT). **b** Surface drawing of AP2M1 colored by its electrostatic potential (blue, positive; red, negative; and white, neutral) with pS616-IRS1 shown as sticks.

The crystal structure of AP2M1 bound to serine-phosphorylated Y^612^XXΦS^616^ motif in IRS1 (pS-IRS1) explains the structural basis of this phospho-switch (Fig. 2). Tyrosine (Y612) and methionine (M615) establish extensive hydrophobic interactions with AP2M1. The hydroxyl group at Y612 forms a hydrogen bond with D176 in AP2M1 (Fig. 2a). YXXΦ IRS1 mutants in which tyrosine was replaced with alanine or phenylalanine showed weakened interaction with AP2M1 in vitro and IR endocytosis was not restored in IRS1-depleted cells. Tyrosine phosphorylation of the YXXΦ motifs is expected to disrupt the IRS–AP2M1 interaction by introducing both static hindrance and unfavorable electrostatic interactions. In IRS1, pS616 is in the vicinity of a positively charged surface on the AP2M1, suggesting that this phospho-serine might participate in favorable electrostatic interactions with this basic region (Fig. 2b).

Consistent with the pS-IRS1–AP2M1 structure, pS-IRS1 binds to AP2M1 with higher affinity, as compared to unphosphorylated IRS1^21^. In addition, pS-IRS1 enhances the tyrosine dephosphorylation of IRS1 by SHP2. These data suggest that ERK-mediated serine phosphorylation of IRS proteins fosters AP2 interaction by directly enhancing the IRS–AP2M1 interaction and indirectly facilitating the SHP2-mediated tyrosine dephosphorylation of IRS, thus promoting clathrin-mediated IR endocytosis. Pharmacological SHP2 inhibition indeed blocks the insulin-stimulated IRS1-AP2 interaction in hepatocytes and delays IR endocytosis. Taken together, these findings establish a direct function of the MAPK pathway in IR endocytosis and, possibly, in metabolic regulation.

In summary, there are two regulatory modules of IR endocytosis: the IRS module and the mitotic checkpoint module (Fig. 1b). The IRS module is activated by the SHP2–MAPK pathway, which is, in turn, activated by insulin signaling. These two modules collaboratively promote the selective endocytosis of activated IR. Our studies suggest that targeting the feedback regulation of IR endocytosis might be beneficial for diabetes treatment. As SHP2 promotes IR endocytosis directly by removing IRS tyrosine phosphorylation and also indirectly by activating the MAPK pathway, inhibition of SHP2 is expected to disrupt the feedback loop, prolong insulin signaling at the PM, and improve insulin sensitivity.

### The use of SHP2 inhibitors for the treatment of cancer and diabetes

Because of its role in growth factor receptor signaling, SHP2 has been implicated in the development of many diseases. Mutations of SHP2 are associated with multiple disorders, most notably Noonan syndrome and LEOPARD syndrome^93^. Somatic SHP2 mutations are also associated with cases of childhood leukemia, including juvenile myelomonocytic leukemia, myelodysplastic syndrome, and acute myeloid leukemia^94,95^. SHP2 overexpression is also causally related to cell proliferation dysfunction. Suppressing SHP2 expression in adult leukemia increases apoptosis and reduces the growth of leukemia cells^96^. Similarly, SHP2 levels are elevated in other types of cancer, including breast and ovarian cancers^97,98^. Due to the association of SHP2 with cancer cell proliferation, SHP2 has emerged as a potential target for cancer therapy.

SHP2 undergoes autoinhibition in the absence of an activator; when not stimulated by insulin, the N-SH2 domain binds the PTP domain and blocks its active site (Fig. 1c)^99^. The small-molecule SHP2 allosteric inhibitor SHP099 takes advantage of this natural regulatory mechanism by interacting with all three domains of SHP2 when it is in the autoinhibited configuration, locking it into the inactive form^57^. SHP099 binds to SHP2 with a high degree of specificity. Strikingly, SHP099 shows no inhibitory activity against SHP1, the closest homolog of SHP2. The effectiveness of SHP099 as an allosteric SHP2 inhibitor makes it a viable option for proof-of-principle studies on SHP2-induced inhibition as a cancer therapy.

Several clinical studies are currently exploring the use of SHP2 inhibitors to treat RTK-mutated cancers (Table 1). SHP099 has been used in conjunction with MEK inhibitors to decrease cancer cell proliferation in multiple types of cancer. This method successfully mitigated the development of the adaptive resistance to MEK inhibition that occurs when MEK inhibitors are used alone^100^. SHP099 is effective in decreasing tumor burden and promoting anti-tumor immunity in mice with grafted colon cancer cells^101^. These findings suggest that SHP099 is a promising candidate for cancer therapy, both as a monotherapy and in conjunction with other agents.

**Table 1:** Ongoing clinical trials and preclinical studies of SHP2 inhibitors.

Inhibitor	Cancer type(s)	Clinical status	Notes^a^
JAB-3068 (Jacobio Pharmaceuticals)	Non-small cell lung cancer (NSCLC) Head and neck cancer Esophageal cancer	Phase 1/2a	NCT03565003
JAB-3312 (Jacobio Pharmaceuticals)	Non-small cell lung cancer (NSCLC) Colorectal cancer Pancreatic ductal carcinoma Esophageal squamous cell carcinoma Head and neck squamous cell carcinoma Breast cancer Other solid tumors	Phase 1	NCT04045496
TN0155^b^ (Novartis)	Non-small cell lung cancer (NSCLC) Esophageal squamous cell cancer (SCC) Head and neck SCC Gastrointestinal stromal tumors	Phase 1/1b	NCT04000529 NCT03114319
RMC-4630 (Revolution Medicines)	Solid tumors (unspecified)	Phase 1b/2	NCT03989115
RLY-1971 (Relay Therapeutics)	Solid tumors (unspecified)	Phase 1	NCT04252339
SHP099	Esophageal cancer cells Hematopoietic cancer cells Colorectal cancer cells KRAS-mutant cancer cells Triple-negative breast cancer	No clinical trials; research involves cell lines and mouse xenografts^57,100,106^.	

^a^https://clinicaltrials.gov/ct2/ (identification number).

^b^Combination with spartalizumab or ribociclib.

Studies on SHP2 and its associated signaling pathways have also revealed its involvement in the regulation of insulin signaling. Patients with LEOPARD syndrome-related SHP2 mutations exhibit resistance to diet-induced obesity, an improved overall metabolic profile, and insulin hypersensitivity^102^. This outcome suggests a possible role for SHP2 inhibition or modulation in treating insulin resistance. Our study has shown that SHP2 inhibitors can be potentially repurposed to treat type 2 diabetes^21^. These results follow the trend shown in previous research indicating a relationship between SHP2 and insulin signaling and suggest SHP2 inhibition as a promising therapeutic method for not only cancer treatment, but also treatment of insulin resistance and diabetes. Cancer and diabetes share common risk factors such as obesity, hyperinsulinemia, and aging. The number of patients suffering from both diseases has increased dramatically in recent years. SHP2 inhibitors may be particularly beneficial to patients who have both diabetes and cancer.

### Targeting IR endocytosis for insulin resistance treatment

**Fig. 3:**
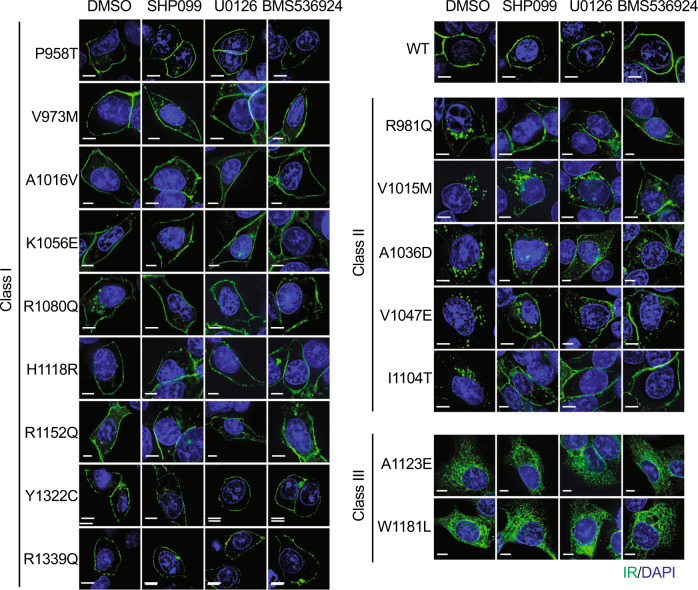
Characterization of IR mutations found in human patients. HepG2 cells expressing IR-GFP wild-type (WT) or mutants were starved for 14 h, treated with the indicated inhibitors for 4 h, and stained with anti-GFP (IR; green; 1181446001, Sigma) and DAPI (blue). SHP099 (SHP2 inhibitor, 10 μM, MedChemExpress), U0126 (MEK inhibitor, 40 μM, Cell Signaling), and BMS536924 (IR kinase inhibitor, 2 μM, Tocris). Scale bar, 5 μm.

**Fig. 4:**
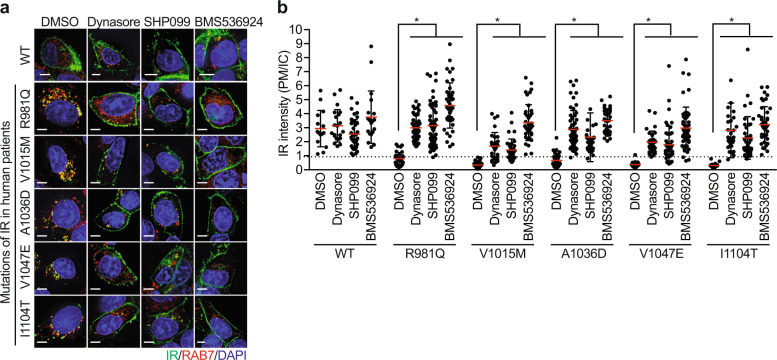
Characterization of class II IR mutations found in human patients. **a** HepG2 cells stably expressing IR-GFP WT or Class II mutants were serum starved for 14 h, treated with the indicated inhibitors for 4 h, and stained with anti-GFP (IR; green) and anti-RAB7 (Red; D95F2, Cell Signaling) antibodies. Dynasore (dynamin inhibitor, 80 μM, Sigma), SHP099 (SHP2 inhibitor, 10 μM, MedChemExpress), and BMS536924 (IR kinase inhibitor, 2 μM, Tocris). Scale bar, 5 μm. **b** Quantification of the ratios of PM and IC IR-GFP signals of the cells shown in **a** (mean ± SD; *p < 0.0001).

Mutations of IR are known to cause inherited severe insulin resistance syndromes^103^, but the mechanisms by which these mutations affect IR function have not been systematically explored. Using the missense mutations in the cytoplasmic region of IR found in patients with severe insulin resistance^103^, we defined three distinct classes of IR mutants based on their subcellular localization in the unstimulated state (Fig. 3 and Table 2). Class I mutants localize to the PM. Class II mutants show reduced signals at the PM and are enriched in RAB7-positive intracellular compartments (Fig. 4). Class III mutants remain in the ER and the Golgi apparatus, indicating that class III mutations affect IR processing and trafficking. The addition of dynasore, a chemical inhibitor of dynamin, elevated the IR level of the class II mutants at the PM (Fig. 4), suggesting that class II mutations cause premature CME of IR prior to insulin stimulation.

**Table 2:** Characterization of IR mutations found in human patients.

Class	Mutation HGVS^a^ nomenclature	Mutation mature, long isoform	Mutation mature, short isoform	Localization^b^	Phenotype
I	P997T	P970T	P958T	PM	Rabson-Mendenhall syndrome
	V1012M	V985M	V973M	PM	Type 2 diabetes
	A1055V	A1028V	A1016V	PM	Insulin resistance
	K1095E	K1068E	K1056E	PM	Type 2 diabetes
	R1119Q	R1092Q	R1080Q	PM	Leprechaunism
	H1157R	H1130R	H1118R	PM	Insulin resistance
	R1191Q	R1164Q	R1152Q	PM	Type 2 diabetes
	Y1361C	Y1334C	Y1322C	PM	Type 2 diabetes
	R1378Q	R1351Q	R1339Q	PM	Insulin resistance
II	R1020Q	R993Q	R981Q	IC	Insulin resistance
	V1054M	V1027M	V1015M	IC	Leprechaunism
	A1075D	A1048D	A1036D	IC	Insulin resistance
	V1086E	V1059E	V1047E	IC	Type 2 diabetes
	I1143T	I1116T	I1104T	IC	Rabson-Mendenhall syndrome
III	A1162E	A1135E	A1123E	ER/Golgi	Insulin resistance
	W1220L	W1193L	A1181L	ER/Golgi	Insulin resistance

*ER/Golgi* endoplasmic reticulum/Golgi apparatus, *IC* intracellular compartment, *PM* plasma membrane.

^a^Human Genome Variation Society, http://www.hgvs.org/rec.html.

^b^The cellular localization of IR-GFP in the basal, unstimulated state.

Treating cells with SHP099 and U0126, a MEK inhibitor, significantly enhanced the PM levels of class II IR mutants, but not those of class I and III IR mutants. These results suggest that targeting IR endocytosis can potentially alleviate insulin resistance in patients with class II IR mutations and possibly in other type 2 diabetes patients. Future studies are required to determine the role of premature IR endocytosis in the pathogenesis of human insulin resistance. Our current study monitored IR endocytosis in HepG2 cells that expressed endogenous IR. Only patients with both alleles of IR mutated displayed insulin resistance phenotypes, whereas their parents, who each had a single mutated allele, did not exhibit these phenotypes. To mimic the situation of the patients, we depleted endogenous IR in HepG2 cells; however, due to high levels of cell death, we could not assess whether the premature endocytosis of IR mutants occurred in the absence of endogenous wild-type IR. It will be interesting to examine the IR PM levels before and after insulin stimulation in patient cell lines that harbor the particular class II mutations and to determine whether inhibitors of SHP2 or the MAPK pathway can recover the IR PM levels and insulin sensitivity.

### Perspective

Our recent studies provide further mechanistic insight into IR endocytosis and raise many interesting questions as follows: (1) the core components of MCC, including MAD2, BUBR1, and CDC20, are assembled onto IR to control its endocytosis in interphase. Is the mitotic module regulated by the SHP2–MAPK pathway during insulin signaling? If it is, what is the main target of SHP2 and MAPK in this module? (2) The fact that an MCC-like complex is assembled onto IR suggests that IR might reciprocally control MCC assembly and spindle checkpoint signaling during cell division. Can insulin and the metabolic environment control genomic stability through IR? (3) How do the two modules–the IRS and mitotic checkpoint modules–cooperate to promote IR endocytosis? How many copies of the AP2 complex, BUBR1, or IRS1/2 are recruited to each IR dimer? Do two modules engage a single AP2 complex? This is theoretically possible because BUBR1 and IRS1/2 do not bind the same site on AP2: BUBR1 binds to AP2B1, and IRS1/2 bind to AP2M1. (4) What is the physiological function of the feedback regulation on human insulin resistance? Can hyperactivation of the MAPK pathway by altered metabolic stress reduce the IR levels at the PM? (5) Are the IR PM levels reduced in human patients harboring class II mutations? Can inhibitors of SHP2 or the MAPK pathway recover the IR PM levels in these patients and improve insulin sensitivity? Future studies aimed at answering these questions will provide further insight into the pathogenesis of insulin resistance.

Type 1 insulin-like growth factor receptor (IGF1R) belongs to the IR family. The intracellular domains of IR and IGF1R share over 80% of amino acid identity. Although IR and IGF1R also share several common adaptors and effectors for downstream signaling pathways, IGF1R only controls cell growth and proliferation, whereas IR controls both cell growth and metabolic homeostasis. The mechanisms by which these two highly homologous receptors achieve different signaling outcomes are largely unknown. In the context of endocytosis, IGF1R binds to IRS1–AP2 but does not have MIM^16,40^, suggesting that the internalization of IGF1R does not involve the mitotic checkpoint module and is solely controlled by the IRS module. Strikingly, Yoneyama et al.^104^ showed that IRS1, but not IRS2, negatively regulates IGF1R endocytosis. Most importantly, the timing of endocytosis after the activation of IGF1R is very different from that of IR. In contrast to activated IR, which is internalized within minutes, activated IGF1R can remain at the PM for over 1 h. Recent cryo-EM structural studies showed that the Γ-shaped asymmetric IGF1R dimer was bound to only one IGF1 molecule, while the T-shaped symmetric IR dimer was bound to multiple insulin molecules^45,105^. Future studies are required to explore whether these important structural differences upon ligand binding affect the endocytosis and downstream signaling of IR and IGF1R.
